# Individual knock out of glycine receptor alpha subunits identifies a specific requirement of *glra1* for motor function in zebrafish

**DOI:** 10.1371/journal.pone.0216159

**Published:** 2019-05-02

**Authors:** Eric Samarut, Domitille Chalopin, Raphaëlle Riché, Marc Allard, Meijiang Liao, Pierre Drapeau

**Affiliations:** 1 Research Center of the University of Montreal Hospital Center (CRCHUM), Department of Neurosciences, Université de Montréal, Montréal, QC, Canada; 2 DanioDesign Inc., Montréal, QC, Canada; 3 Modelis Inc., Montréal, QC, Canada; 4 UnivLyon, ENS de Lyon, Université de Lyon, CNRS UMR5239, INSERM U1210, Lyon, France; University of Colorado Boulder, UNITED STATES

## Abstract

Glycine receptors (GlyRs) are ligand-gated chloride channels mediating inhibitory neurotransmission in the brain stem and spinal cord. They function as pentamers composed of alpha and beta subunits for which 5 genes have been identified in human (GLRA1, GLRA2, GLRA3, GLRA4, GLRB). Several *in vitro* studies showed that the pentameric subtype composition as well as its stoichiometry influence the distribution and the molecular function of the receptor. Moreover, mutations in some of these genes are involved in different human conditions ranging from tinnitus to epilepsy and hyperekplexia, suggesting distinct functions of the different subunits. Although the beta subunit is essential for synaptic clustering of the receptor, the specific role of each alpha subtype is still puzzling *in vivo*. The zebrafish genome encodes for five glycine receptor alpha subunits (glra1, glra2, glra3, glra4a, glra4b) thus offering a model of choice to investigate the respective role of each subtype on general motor behaviour. After establishing a phylogeny of GlyR subunit evolution between human and zebrafish, we checked the temporal expression pattern of these transcripts during embryo development. Interestingly, we found that *glra1* is the only maternally transmitted alpha subunit. We also showed that the expression of the different GlyR subunits starts at different time points during development. Lastly, in order to decipher the role of each alpha subunit on the general motor behaviour of the fish, we knocked out individually each alpha subunit by CRISPR/Cas9-targeted mutagenesis. Surprisingly, we found that knocking out any of the alpha2, 3, a4a or a4b subunit did not lead to any obvious developmental or motor phenotype. However, *glra1*-/- (*hitch*) embryos depicted a strong motor dysfunction from 3 days, making them incapable to swim and thus leading to their premature death. Our results infer a strong functional redundancy between alpha subunits and confirm the central role played by *glra1* for proper inhibitory neurotransmission controlling locomotion. The genetic tools we developed here will be of general interest for further studies aiming at dissecting the role of GlyRs in glycinergic transmission *in vivo* and the *hitch* mutant (*hic*) is of specific relevance as a new model of hyperekplexia.

## Introduction

Glycine receptors (GlyRs) are ligand-activated chloride channels mediating fast inhibitory neurotransmission in the adult brain stem and spinal cord. GlyRs depict a modular structure with a N-terminal extracellular glycine binding domain followed by four transmembrane domains (M1 to M4) at their C-terminal part and a long intracellular loop between M3 and M4. GlyRs function as pentamers composed of different beta and/or alpha subunits that form a pore letting chloride ions flow through the cell membrane once opened. Five GlyR subunit genes have been referenced in mammals consisting of 4 alpha (GLRA1, GLRA2, GLRA3, GLRA4) and one beta subunit (GLRB) and these genes can be considered as paralogs. Of note is that GLRA4 is considered as a pseudogene in human. Interestingly, chloride conductance and other channel molecular features are influenced by the subunit composition as well as their very diverse stoichiometry (3α1:2β, 2α1:3β, or 1α1:4β) [1–4]. Although heteromeric channels are thought to mediate most of the glycinergic neurotransmission in the adult [5–8], GlyRs can also form homomeric pentamers of alpha subunits only. These homomeric channels are believed to be extra-synaptic [9, 10] since only the beta subunit is able to bind to gephyrin, an anchoring protein necessary for GlyR localization at the synapse [11, 12].

In the last decades, several studies attempted to unravel the specific role of each GlyR subunit *in vivo*. To do so, mutant mice lacking the expression of either the Glra1, 2 or 3 alpha subunits have been generated. Interestingly, although *Glra1-/-* mice depict motor dysfunction reminiscent to hyperekplexia, *Glra2-/-* and *Glra3-/-* do not depict motor impairment. However, *Glra3* seems to play a role in inflammatory pain sensitization [13–15]. More recently, zebrafish became a convenient vertebrate model to study motor coordination impairment *in vivo*. As a result of a third-round of whole genome duplication in the teleost lineage, teleost fishes doubled many of their genes [16]. Thus the zebrafish genome encodes for seven GlyR subunit genes (five alpha: *glra1*, *glra2*, *glra3*, *glra4a*, *glra4b* and two beta: *glrba*, *glrbb*) that are the result of secondary loss of some duplicated paralogs [17–19]. Knockdown experiments targeting different individual subunits shed light on their specific role. Indeed, whereas knocking down *glra1* and *glrbb* induces spasticity, morpholino injection against *glra4a* (also known as the embryonic alpha 2 subunit at that time) perturbed interneuron neurogenesis in the spinal cord by modifying the transcriptome of neural stem cells [20–22]. Interestingly, a knockout mutant of *glrbb* was generated in zebrafish (*beo*) depicting bilateral contraction of the trunk and is a model of hyperekplexia [23]. Of note is that the knockout or knockdown of the same gene (*glrbb*) does not lead to the same phenotype (spasticity vs bilateral contraction), thus suggesting that a complete loss-of-function *versus* a hypomorphic allele could lead to different phenotypes. So far, no knockout mutant has been generated for the five GlyR alpha subunits. In this work, we systematically knocked them out individually by CRISPR/Cas9 in order to investigate the consequences of the lack of each alpha subunit on the motor behaviour of the fish.

First, we drew a new phylogeny of the zebrafish GlyR genes to ensure that the terminology (that has changed a lot within the last 20 years) infers a correct orthology between mammalian and zebrafish GlyR genes. Next, we followed the temporal expression of each GlyR subunit during zebrafish embryonic development in order to see if any specificity in timing of expression could be observed between subunits. Lastly we knocked out one by one each of the five alpha subunits and monitored the general development as well as the larval motor ability. We showed that none of the glra2, glra3, glra4a and glra4b mutants depict any obvious motor phenotype and they all survive loss-of-function homozygosis until adulthood. Interestingly, only the *glra1* mutant (*hitch*) displayed a motor phenotype with defects in motor coordination of the tail, thus preventing correct swimming and leading to a premature death. Our work here sheds new light on the specific requirement of each alpha subunit *in vivo* and provides a new and convenient *in vivo* model for the study of hyperekplexia.

## Materials & methods

### Fish husbandry

Wild-type zebrafish (*Danio rerio*) were reared at 28.5°C, kept under a 12-hour dark, 12-hour light cycle and staged as described previously [24]. They were bred according to standard procedures [25]. All experiments were performed in compliance with the guidelines of the Canadian Council for Animal Care and conducted at the Centre de recherche du centre hospitalier de l’Université de Montréal (CRCHUM) and approved by the Institutional Committee for the Protection of Animals of the CRCHUM (approval # N15018PMDz). All experiments were performed on sexually undifferentiated zebrafish larvae between 1–7 days post-fertilization (dpf). Humane endpoints were in place during the study and all animals were monitored and assessed daily for well-being as per guidelines established by the Canadian Council of Animal Care committee at the CRCHUM. Behavioral signs of poor health in adult animals necessitating euthanasia included an inability to feed and swim. Physical abnormalities were also monitored daily and adult animals displaying a distended abdomen, skin ulcerations/wounds and skeletal deformities were euthanasia immediately by anesthetic overdose and rapid chilling.

### Phylogeny

The evolutionary history of the GLR family including GLRA and GLRB was investigated through phylogenetic analyses and synteny. Protein sequences were retrieved from NCBI (Genbank (https://www.ncbi.nlm.nih.gov/genbank/) and Ensembl (http://www.ensembl.org/index.html) databases. Sequences were then aligned using clustalo tool[26] with seaview 4.5.4 interface [27]. Phylogenetic trees were reconstructed using Raxml-ng (DOI:10.5281/zenodo.593079) with options “—all—model LG+G”. Finally, graphical display of the tree was done using Figtree v1.4.3 software (http://tree.bio.ed.ac.uk/software/figtree/). Conservation of syntenic block including GLR genes was determined using Genomicus v92.01[28]. To resolve the GLRB history and duplication, sequences were further investigated using blast analyses. The human GLRB amino acid sequence was used as a query to perform tblastn on all available Ensembl (http://www.ensembl.org) teleost genomes as well as the spotted gar and coelacanth.

### Reverse transcription and polymerase chain reaction (RT-PCR)

Reverse transcription was performed from 1μg of total RNA using the superscript VILO reverse transcription mix (Invitrogen). PCR was performed on 1 μL of cDNA using regular GeneDirex Taq polymerase (FroggaBio) using gene-specific primers (sequences upon request).

### CRISPR/Cas9 targeted mutagenesis

All guide RNAs were designed using the online tool CRISPRscan. The list of guide RNA sequences as well as the targeted exon for each gene is indicated in the Table 1 below.

**Table 1:** *CRISPR/Cas9 molecular toolkit for individual knockout of GlyRs*.

Gene	Exon	Genomic target 5’ to 3’ (PAM)	Guide RNA sequence (mismatch)
*glra1*	5	GACAGCAGTGGAATGACCCT(CGG)	G(G)CAGCAGTGGAATGACCCT
*glra2*	4	GCTGGATAAAGGGCCCGTTC(AGG)	G(G)TGGATAAAGGGCCCGTTC
*glra3*	4	GGCAGAAGTGGAACGACCCC(CGG)	GGCAGAAGTGGAACGACCCC
*glra4a*	5	TGATAACCCTGTGCAGGTTG(CGG)	(GG)ATAACCCTGTGCAGGTTG
*glra4b*	7	AGGATCACTGTTAAAAGGCT(GGG)	(GG)GATCACTGTTAAAAGGCT

Synthesis of gRNA, Cas9 and NANOS-Cas9 mRNA was performed as described by [29]. Wild-type embryos were collected for microinjection. A 1nL drop of a mix of 100 ng/μL of Cas9 mRNA and 30 ng/L of gRNA was injected into one-cell stage embryos.

### Genotyping

3 days old embryos were transiently anesthetised in tricaine methanesulfonate (MS222) at a final concentration of 160 mg/L and a tiny piece of caudal fin was cut using a microblade. Adult fish were anesthetised in tricaine methanesulfonate at a final concentration of 160 mg/L and a small piece of the caudal fin was cut with a sharp blade. The fish were immediately put back in fresh water in isolated tanks. Genomic DNA extraction was performed in 10μL (embryos) or 20 μL (adult) of 50 mM NaOH. The samples were boiled for 10 minutes and 1/10 volume of 100 mM Tris-HCl pH 8 was added to buffer the reaction, as described in [29]. Primers were designed using the Universal Probe Library Assay Design Center (Roche). The PCR reactions was performed as described by [29].

### Swimming activity monitoring and imaging

At 7 days post-fertilization (dpf), larvae were transferred individually into a 96-well plate and activity was recorded over 4h/4h light-dark cycles using Basler GenIcam camera and DanioVision recording chamber (Noldus). Analysis was performed using the Ethovision XT 12 software (Noldus) to quantify the distance swam. For slow-motion imaging of touched-evoked response, *glra1*+/+ or -/- larvae were placed in a 10mm petri dish and the touch-evoked response was recorded at 120 fps using an iPhone 6 mounted on a iDu Optics cast from LabCam. Raw values are available in the Supporting Information (S1 Table).

### Morphological measurement

Glra1+/+ and -/- larvae were anaesthetized and dorsal and lateral images of the whole body were acquired using the same parameters for all larvae. Body length and eye size using the Danioscope software. Body length was measured from the anterior tip to the caudal peduncle. Eye size was measured by manually specifying the eye boundaries. Raw values are available in the Supporting Information (S2 Table).

## Results

### Evolutionary history of zebrafish glycine receptor subunits

**Fig 1:**
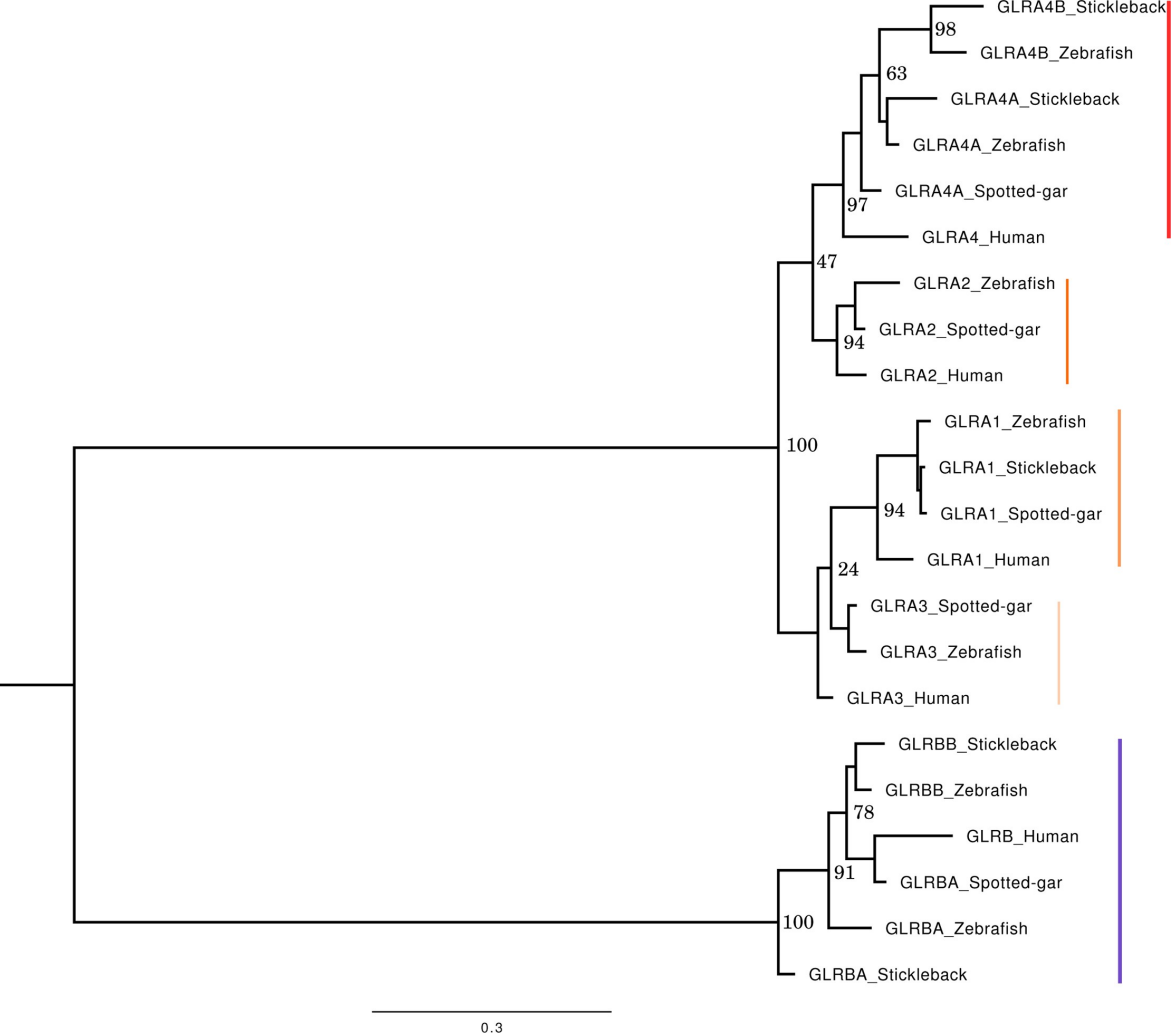
Phylogenetic reconstruction of the GLR family. Reconstruction from amino acid alignment containing 370 sites. The bottom bar represents the scale for genetic changes (number of substitutions per site). Numbers at the nodes represent bootstrap statistical values. Note that the separation between GLRA2/4 and GLRA1/3 is strongly supported. However, bootstraps are weak at the node separating GLRA2 from GLRA4 and at the node separating GLRA1 from GLRA3. Multiple factors can affect supporting values, including sequences with high similarities (which is the case here). Each GLRA gene is highlighted with a reddish sideline, while the blue sideline indicates GLRB genes.

Five alpha (*glra1*, *glra2*, *glra3*, *glra4a*, *glra4b*) and two beta (*glrba*, *glrbb*) subunits have been cloned in zebrafish and their terminology has changed multiple times in the last 20 years. As a result, the field is lacking an evolutionary history of GlyR subunit genes. Low and colleagues recently published the GLR phylogeny including human, mouse and zebrafish sequences [30]. This work highlights the two GLRB and GLRA4 duplications in zebrafish compared to human. To further investigate the evolutionary history of the GLR family in vertebrates and better characterize the duplication events, we investigated the genomic landscapes of the genes. We first collected protein sequences from human, two teleosts (zebrafish and stickleback) as well as the actinopterygian spotted gar, a teleost outgroup to better date the duplication event. GLRA and GLRB form two distinct clades on phylogenetic reconstruction (Fig 1). Focusing on GLRA, two main branches separate GLRA2 and 4 on one side and GLRA1 and 3 on the other. The four genes contain one human sequence and one spotted gar sequence. GLRA1, 2 and 3 also harbor a single copy per teleost. GLRA4 contains two sequences of each teleost, showing a teleost-specific duplication of the GLRA4 gene, dating after the divergence between the spotted gar and teleost species. Note that GLRA2 sequence is missing in stickleback. Blast analysis using the zebrafish GLRA2 on the stickleback genome reveals that the closest sequence is GLRA4B, but no GLRA2 sequence could be identified.

The human and the spotted gar also harbor a single copy of GLRB gene, respectively named GLRB and GLRBA in the database. Both zebrafish and stickleback have two GLRB genes, named GLRBA and GLRBB, indicating once again a teleost-specific duplication.

**Fig 2:**
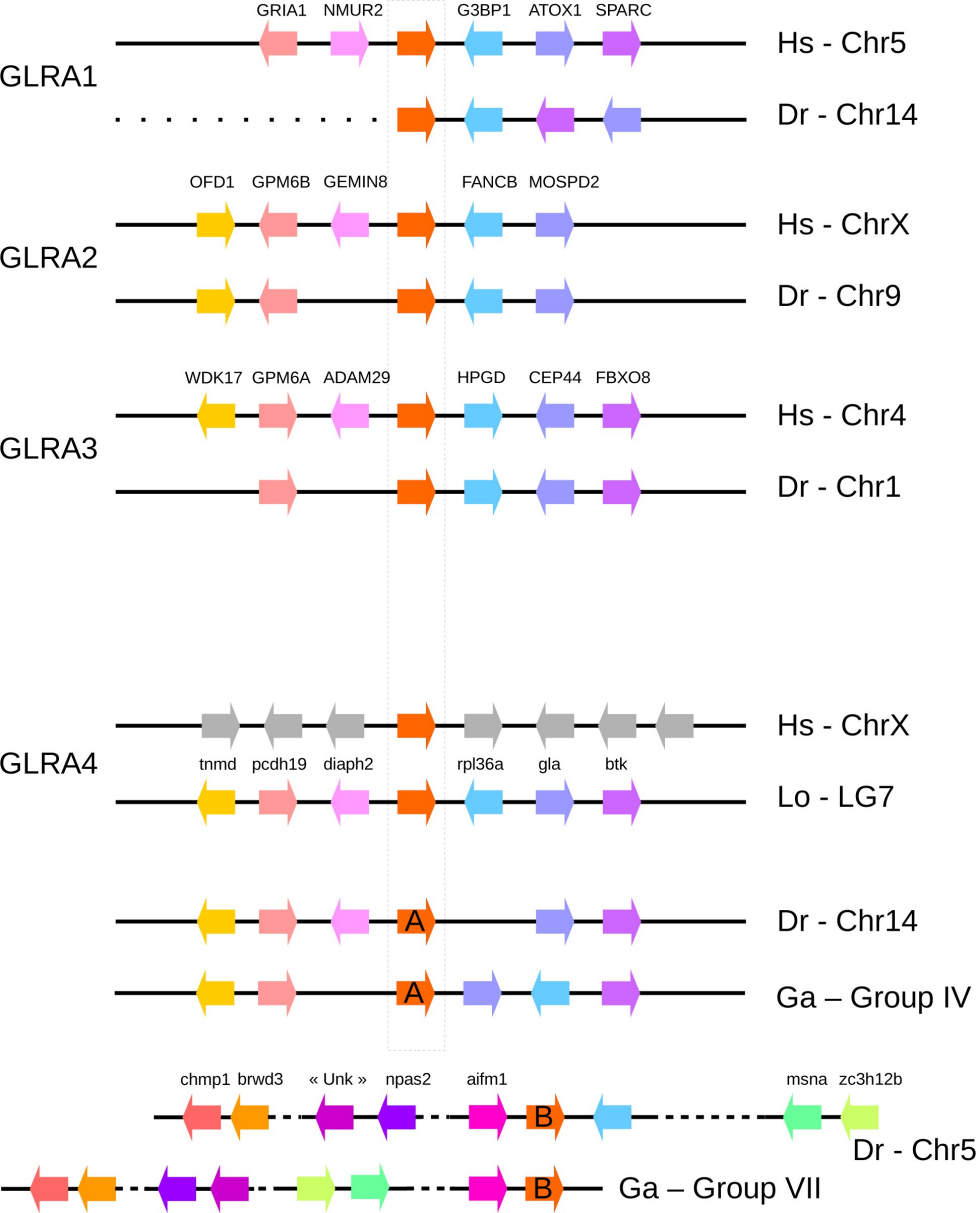
Genomic block conservation between human, spotted gar, stickleback and zebrafish. Black lines represent a genomic region and colored arrows represent oriented genes. GLR genes are indicated by red arrows. Same genes are indicated by similar color in the different species. Spotted gar genomic regions were added for GLRA4 and GLRBB to better understand the duplication events. Hs, Homo sapiens; Lo, Lepisosteus oculatus; Dr, Danio rerio; Ga, Gasterosteus aculeatus; Chr, Chromosome.

To better characterize the GLRA4 and GLRB duplications, we looked at their synteny. In other words, we looked at the conservation of gene orders between human and teleost species. Despite a few rearrangements, GLRA1, 2 and 3 show a high conservation of blocks in vertebrate lineages (Fig 2). It is however much more complicated for GLRA4 and GLRB. Regarding GLRA4, no conservation at all was found between the human region and actinopterygian (teleost and spotted gar). The region of the GLRA4A in teleost resembles the one in spotted gar, with the exception that *diaph2* gene is lost in stickleback and the zebrafish *rpl36a* is located in GLRA4B region. GLRA4B region contains really different genes. These results may indicate first that GLRA4A and B are teleost gene-specific duplications of GLRA4 (synteny would be conserved between the two genes in case of genome or chromosome duplication) and second that GLRA4A is the ancestral copy (as synteny environment resembles that of the spotted gar), as supported by the phylogenetic reconstruction. The presence of introns in GLRA4B excludes the hypothesis of a retrocopy.

The region of the GLRB gene is very conserved between human and spotted gar. *Gria2* and *Tmem144* positions are conserved in the zebrafish region, while none of these genes are found in the stickleback region. The GLRBB region is more conserved in the two teleosts. The observations indicate that the whole region of GLRB may have been duplicated in teleosts, followed by multiple losses around GLRBA.

### Differential temporal expression of zebrafish GlyR subunits

**Fig 3:**
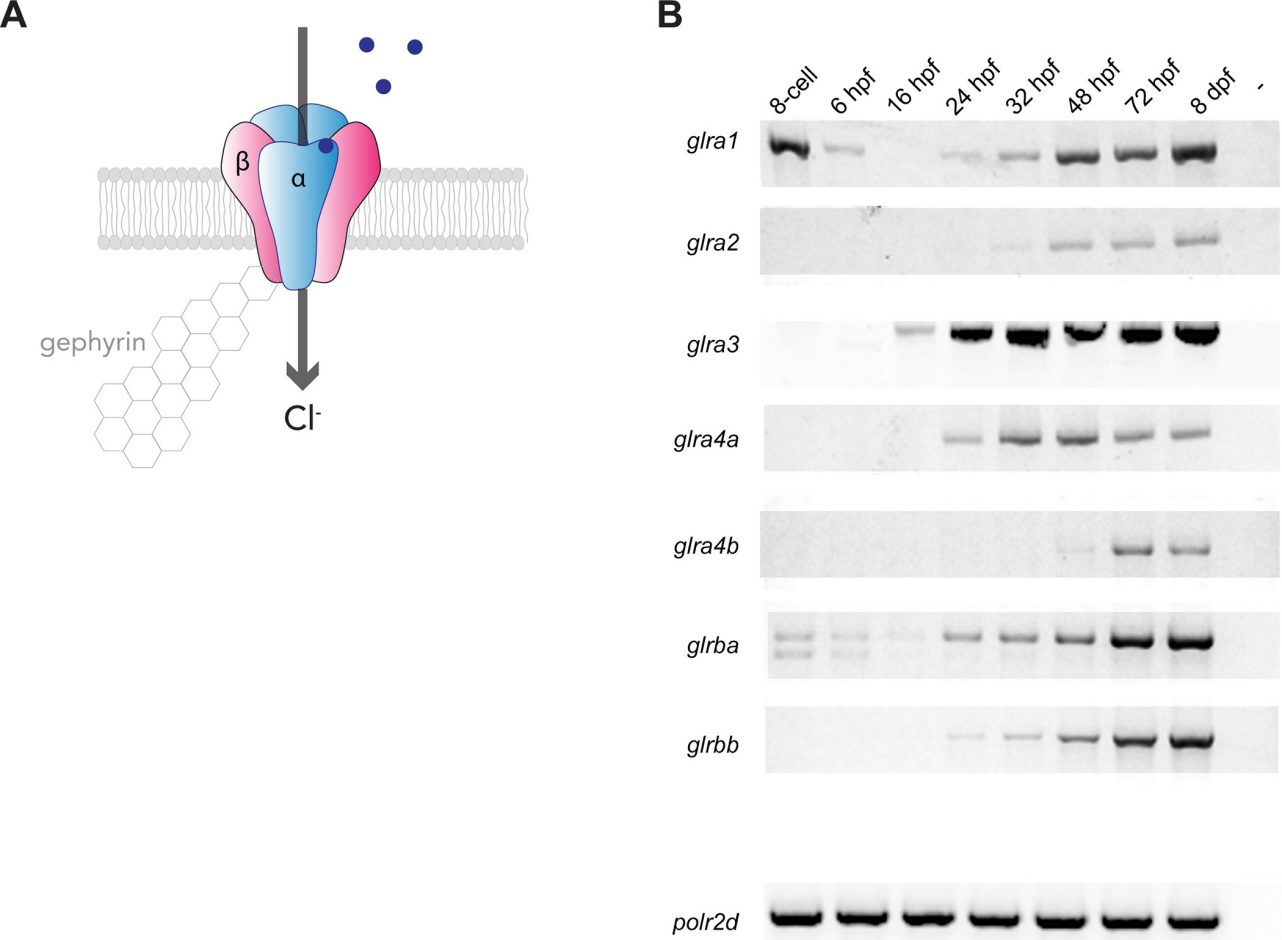
Glycine receptor subunits temporal expression. A, Scheme of a (3α,2β) glycine receptor. Beta subunits ensure synaptic anchoring through binding to cytoplasmic gephyrin proteins. Glycine binding triggers the opening of the chloride channel. B, RT-PCR of each glycine receptor subunits from whole embryo RNA extracts at different developmental stages. Polr2d is used as a reference gene.

We first checked the temporal expression pattern of each of the seven zebrafish GlyR subunits by RT-PCR at different time points during embryo development from 6 hours post fertilization (hpf) up to 8 days. We also checked their expression at an earlier time point (8-cell stage) at which the load of mRNAs in the embryo is exclusively maternally transmitted [31]. (Fig 3A and 3B). A recent study analysed the relative expression by relative quantitative PCR [30] but we aimed at checking the absolute expression over time by RT-PCR. Interestingly, we found that *glra1* is the only alpha subunit transcript detected in the first cells of the embryo and its expression drops after the activation of the zygotic transcription (at the mid-blastula stage, from 3 hpf). *Glra1* starts to be expressed again at 24 hpf and its expression increases with time. We also detected a faint expression of *glrba* at the 8-cell stage that vanishes at 16 hpf. As a result, glra1 and glrba are the only two GlyR subunits that we could detect from the maternal RNA pool. At 16 hpf, *glra3* appears to be the main subunit being transcribed and its expression increases rapidly from 24 hpf. Concomitantly, *glra1*, *glra4a* and *glrba* expression is detectable from 24 hpf and they become stronger with time. Surprisingly, *glra2*, whose orthologous gene *GLRA2* is expressed embryonically in mammals, is only detectable in the zebrafish embryo from 32 hpf and its temporal expression follow the one of *glrbb* whose starts at 32 hpf and increases with time. Lastly, *glra4b* expression is faintly detectable at 48 hpf and starts to be expressed more robustly from 3 dpf. Thus these results show that they are some differences on the temporal expression of GlyR subunits during development. Altogether these results show that the different GlyR subunits depict a different temporal expression throughout embryo development but that all subunits are robustly expressed from 72 hpf.

### Individual knockout of each GlyR alpha subunit

**Fig 4:**
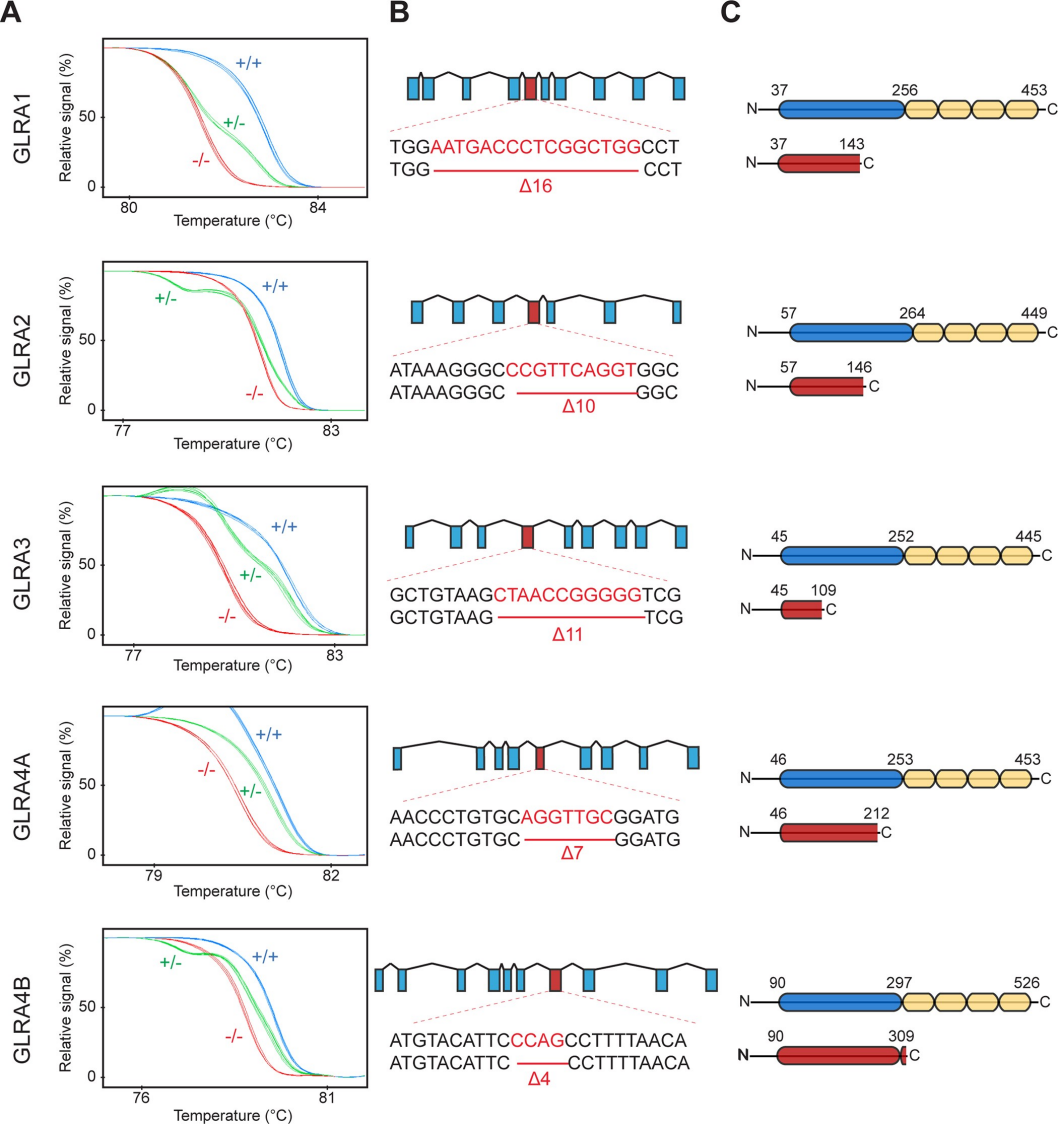
Systemic knockout of each individual glycine receptor alpha subunit. A, High Resolution Melting profiles of knock-out lines discriminating between wild-type (+/+), heterozygous (+/-) and homozygous (-/-) siblings. B, Genomic scheme showing the targeted exon for each knockout lines as well as the selected frameshifting deletion. C, Protein scheme showing the truncated protein caused by the mutation (in red). The glycine binding domain (in blue) and the transmembrane domains (in yellow) are shown in the wild-type protein (top).

In order to shed light on the specific role of each GlyR alpha subunit, we designed specific guide RNAs for targeted mutagenesis of all five of the zebrafish alpha subunits (Fig 4). The targeted genomic loci encoded the glycine binding domain (or TM1) in order to induce a complete loss-of-function. We raised the F0 generations of CRISPR/Cas9 injected embryos to adulthood and screened them for transmission of mutations to the F1 generation. We noticed that CRISPR/Cas9 injection targeting *glra1* induced a high level of mortality in the injected embryos and we were not able to identify a positive founder for this gene. Thus, we injected a new batch with the Cas9-nanos construct that encodes for a germline-targeted Cas9 endonuclease, thus reducing somatic mutation and increasing viability of F0 injected embryos [32]. For each gene, we selected by high-resolution melting assay a frame shifting deletion leading to a premature stop codon (Fig 4A and 4B). These mutations are expected to lead to non-functional proteins lacking half of the glycine binding domain and/or all transmembrane domains (Fig 4C). All five lines were raised as heterozygous until adulthood for further in-cross.

### Specific requirement of *glra1* for motor function

**Fig 5:**
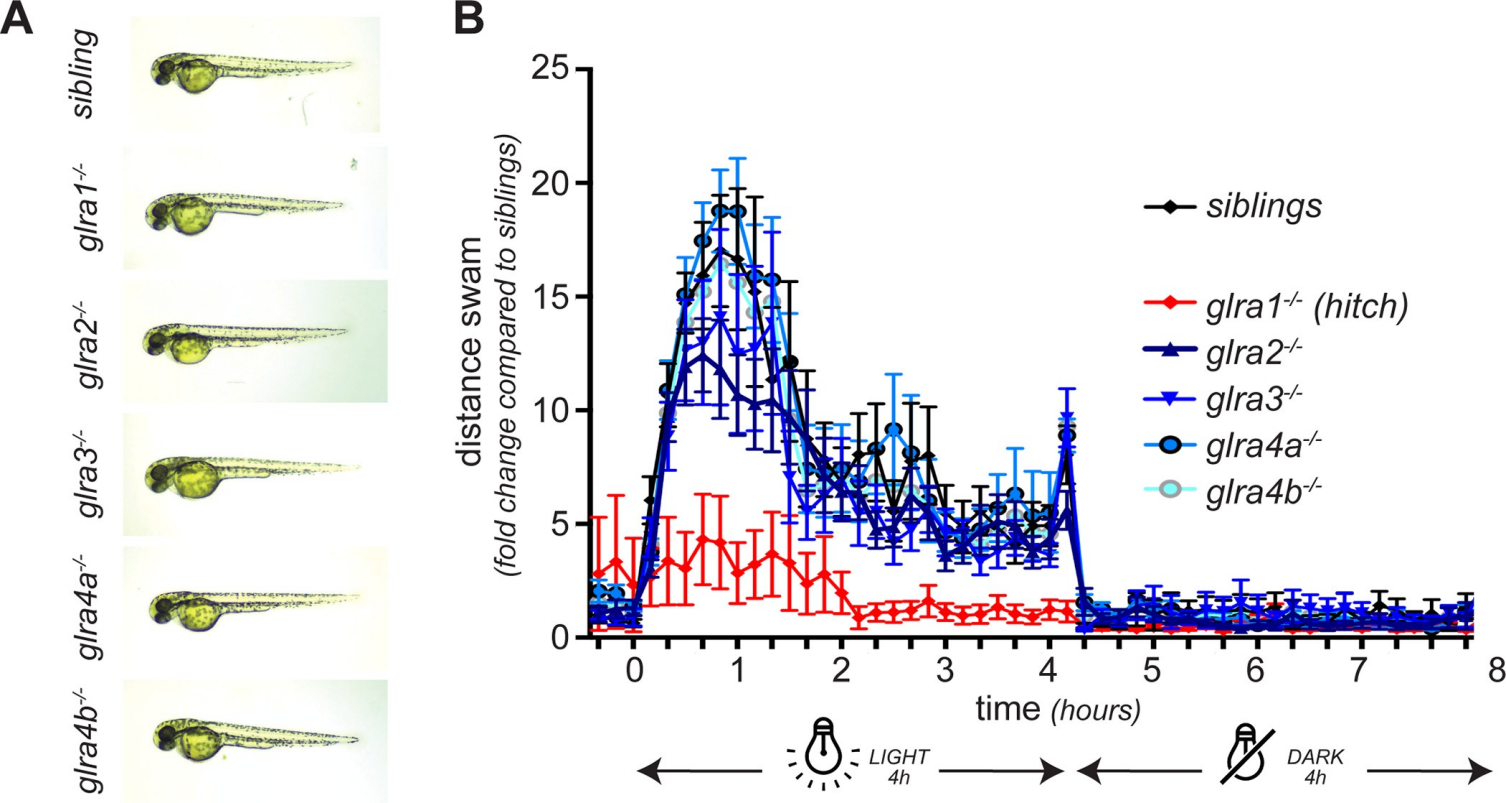
Morphology and swimming activity of GlyR alpha subunit knockouts. **A,** For each knock-out, the embryos developed normally and no abnormal morphology was observed at 48 hpf. **B,** However, when their swimming activity was assessed at 5 dpf during 4h/4h dark/light condition, hic (glra1-/-) mutant larvae depicted a decreased activity with no startle response to light compared to their siblings. No difference was observed for the other knockout lines.

In order to assess the effect of each individual knockout on the general development of the embryo and the motor behaviour of the larvae, we followed the development of embryos obtained from in-crosses of heterozygous parents for each of the five mutant lines generated. We observed no obvious morphological abnormalities for any of the five mutant lines at 2 dpf (Fig 5A). We then tested the swimming ability of mutant larvae at 7 dpf by monitoring their swimming distance during a 4h/4h light/dark cycle in an automated recording chamber (Fig 5B). Interestingly, although we did not notice any difference between siblings and *glra2-/-*, *glra3-/-*, *glra4a-/-* and *glra4b-/-*, we found that *glra1-/-* (*hic*) are hypoactive. Indeed, whereas siblings respond to light with an increased activity, *hic* larvae barely show an increase of activity and stay hypoactive all along the swimming assay (Fig 5B). Of note is that no difference was observed between the other mutants regarding the distance swam, the maximum acceleration or any other monitored parameter.

### *Hitch (hic)* mutant larvae depict a motor coordination defect with a reduced body size

**Fig 6:**
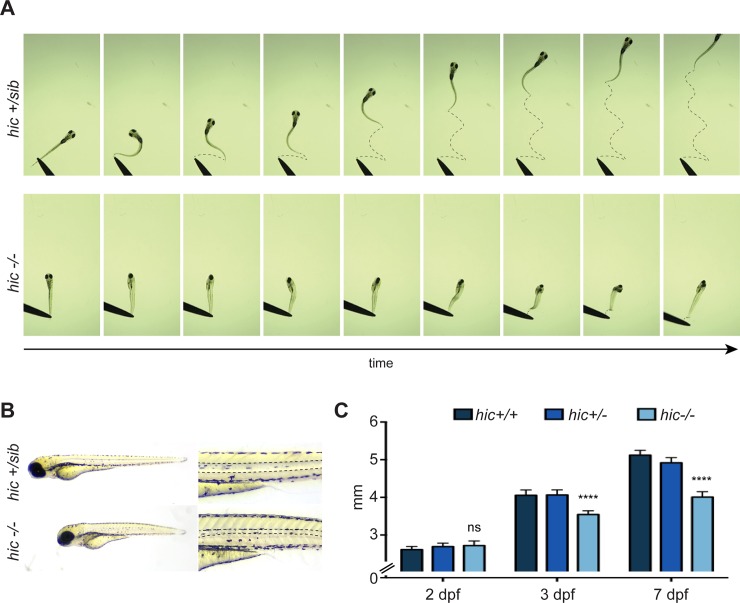
Hic mutants display motor coordination dysfunction. **A,** Touch-evoked escape response of hitch mutants versus their siblings at 7 dpf show a dysfunction of motor coordination of hic-/- larvae. Indeed, hic-/- larvae are not able to bend their tail in a coordinated manner in order to initiate an escape swimming response (see S1 and S2 Movies for full recordings). **B,** Hic-/- larvae at 7 dpf show a reduction of the trunk size as well as defects of the notochord and a change in birefringence of trunk muscles. **C,** Measurement of the trunk size at different age shows that the reduction in trunk length starts at 3 dpf and aggravates at 7 dpf presumably due to the motor dysfunction.

Since our swimming assay showed a defect in the swimming behaviour of *hic* larvae, we looked into more detail about their motor coordination. To do so, we monitored at a high-frame per second rate (120 fps) the touch-evoked escape response of *hic*^*-/-*^ versus siblings (*hic*^*+/sib*^) (Fig 6A). Whereas sibling larvae escaped vigorously from the touch by bending smoothly their tail from one side to another, *hic* mutant larvae failed to coordinate motor escape behaviour (Fig 6A, S1 and S2 Movies). Indeed, following the touch, mutant larvae bend their trunk and undergo jerks of the tail as well as hitching of their movement, corresponding to pauses disrupting the smooth progression of the escape movement, as described earlier by Ganser and colleagues [33]. We therefore nicknamed glra1-/- as *hitch* (*hic*) mutant. The phenotype is different from the bilateral contraction observed in *beo* mutant (*glrbb-/-*) and they often succeed to bend their tail once following the touch but failed to escape. Although *hic* mutants are indistinguishable from their siblings at 2 dpf, they start to depict a reduced trunk size from 3 dpf that became more obvious with time (Fig 6B and 6C). Moreover, we noticed defects of the notochord and atrophy of the somite muscles (Fig 6B). These morphological abnormalities are reminiscent to the ones observed in *beo* mutants and are likely to be secondary effects due to the mechanical stress caused by the motor coordination defect. As for *beo*, *hic* mutants die prematurely after one week of age, presumably being unable to swim and feed.

## Discussion

In this work, we aimed at investigating the specific function during locomotion of each glycine receptor alpha subunits *in vivo*, taking advantage of the zebrafish embryo. Unexpectedly, we noticed a strong functional redundancy between glra2, a3, a4a and a4b different subunits, with alpha 1 being the only one specifically required for correct glycine inhibitory neurotransmission involved in motor function. This is consistent with data obtained from mice models lacking the alpha 1 subunit that depict a motor dysfunction reminiscent to hyperekplexia [13]. The fact that knocking-out the other alpha subunits did not lead to a major motor phenotype is also consistent with the fact that glra2-KO and glra3-KO mice do not display motility defects but rather inflammatory and sensory dysfunctions [14, 15]. At this juncture, our new genetic lines bearing homozygous knockout mutations in each individual a2, a3, a4a and a4b subunit could be of interest for further studying the role of glycinergic transmission in a broad variety of physiological processes, especially during development due to the ease of studying the zebrafish embryo. Moreover, although beyond the scope of the present study, these lines could be further used to characterize the electrophysiological involvement of each alpha subunit on the channel activity. This would allow to further describe the specific function of each subunit at the molecular level. Moreover, we believe that the *hitch* mutant lacking the expression of the alpha 1 subunit is an interesting model to complement the genetic tools already available to investigate hyperekplexia and other glycine-related disorders. Indeed, one third of the patients with hyperekplexia carry a loss-of-function mutation in the alpha 1 subunit (*GLRA1*) of the glycine receptor and such a genetic condition has not been previously modeled in zebrafish. Thus, our *hitch* (*hic*) mutant complements the library of zebrafish genetic models with glycinergic transmission defects that are *bandoneon* mutants (*beo*) knocked-out for *glrbb* [23] and *shocked* mutants (*sho*) knocked-out for glycine transporter 1 (*glyT1*) [34].

Interestingly, *glra1* and *glrbb* are co-expressed in reticulospinal neurons in zebrafish [23]. These big neurons are responsible for a very fast escape reflex in teleosts and amphibians, thus consistent with the fact that both *beo* (*glrbb*-/-) and *hic* (*glra1*-/-) depict defects in their escape motor coordination. Moreover, our results showing that *hic* mutants display a defective escape response are consistent with a role of the alpha 1 subunit in the glycinergic neurotransmission within these neurons (i.e Mauthner cells). However the fact that they do not display the exact same defects (bilateral contractions *versus* hitching) suggest that they might play specific functions at the molecular level. The zebrafish has become an attractive model to study human disorders by complementing the genetic aspects of human diseases studied from both invertebrates (nematodes and flies) and mammalian models. As a result, we believe that our *hitch* mutant could be useful for further discovery of small molecules counter-acting glycine neurotransmission defects in a therapeutic endeavour.

Although our findings are consistent with previous work from knocked-out mouse models, they surprisingly do not recapitulate the morpholino-induced GlyR knockdown phenotypes observed in zebrafish. Indeed, we did not observe any swimming defect or developmental abnormalities in *glra3*-KO and *glra4a*-KO, as is the case after microinjecting morpholinos targeting these subunits [35]. Moreover, we were not able to detect any defect of the neural progenitor population in *glra4a*-KO fish although this phenotype was described earlier using morpholino-knockdown strategy [21]. These observations raise the idea that the transient knockdown of a gene expression can induce a different phenotype than a complete knockout of the same gene. One likely scenario at the origin of such a difference is that complex compensatory genomic networks could be recruited following gene knockout but not after partial knockdown, in order to buffer against steady deleterious mutations [36].

Finally, our results showing a large functional redundancy, at least at the level of motor function, between glycine receptor alpha subunits open an interesting discussion regarding the evolutionary advantages of glycine receptor subunit gene duplication. Indeed, gene duplication is usually considered to participate in the acquisition of new phenotype or in fine-tuning of physiological processes during evolution [37]. For the case of the zebrafish, the fact that all five alpha subunits are expressed during development suggests that they still carry required functions since they could have evolved as pseudogenes in the opposed scenario. As a matter of fact, one could wonder what is the beneficial physiological interest of those gene copies in the light of genomic evolutionary constraints. One reasonable explanation would be that although the GLRA1 subunit is indispensable for motor function and therefore for larvae survival, the other subunits could play more subtle functions that, even though not crucial for larvae thriving, still constitute an evolutionary advantage.

## Supporting information

S1 Movie Time-lapse recording (120 fps) of a touch-evoked response of a hic+/sib 7 dpf larvae. (MP4)

S2 Movie Time-lapse recording (120 fps) of a touch-evoked response of a hic-/- 7 dpf larvae. (MP4)

S1 Table Raw values of swimming activity. (XLSX)

S2 Table Raw values of morphological measurements. (XLSX)
